# Primary epithelioid rhabdomyosarcoma of the stomach: a case report and review of literature

**DOI:** 10.1186/s13000-019-0917-y

**Published:** 2019-12-23

**Authors:** Yangkun Wang, Pei Guo, Zhishang Zhang, Runde Jiang, Zuguo Li

**Affiliations:** Department of Pathology, Shenzhen Hospital, Southern Medical University, 1333 Xinhu Road, Bao’an District, Shenzhen City, 518110 China

**Keywords:** Gastric tumor, Epithelioid rhabdomyosarcoma, Clinicopathological features, Immunohistochemistry

## Abstract

**Background:**

Epithelioid rhabdomyosarcoma is a rare tumor that generally occurs in the bladder, the parotid gland, or the skin of the neck. We describe an unusual case of primary epithelioid rhabdomyosarcoma of the stomach and review the literature.

**Case presentation:**

A 64-year-old woman presented with a lesion at the gastroesophageal junction. Histopathological examination showed irregularly sized round cells with low cytoplasmic content and eccentric nuclei. Mitotic figures were present. Fibrovascular septa and areas of necrosis were observed between tumor cells. Tumor cells were strongly positive for MyoD1, desmin, and myogenin, and weakly positive for actin, CD56, and PGP9.5. The ki-67 index was ≥90%.

**Conclusions:**

Primary epithelioid rhabdomyosarcoma of the stomach is extremely rare. Better awareness of this entity is necessary for early diagnosis and treatment.

## Background

Rhabdomyosarcomas are a group of soft tissue malignant tumors characterized by skeletal muscle formation. The World Health Organization (WHO) classification includes four types: embryonal rhabdomyosarcoma, alveolar rhabdomyosarcoma, spindle cell rhabdomyosarcoma, and pleomorphic rhabdomyosarcoma [1–3]. Epitheloid rhabdomyosarcoma (ERMS) is a recently recognized rare variety that has been previously reported in the urothelium of the bladder, the parotid gland, and the skin of the neck [4–6]. We report an unusual case of ERMS of the esophagogastric junction and discuss the differential diagnosis. We also briefly review the literature.

## Case presentation

**Fig. 1:**
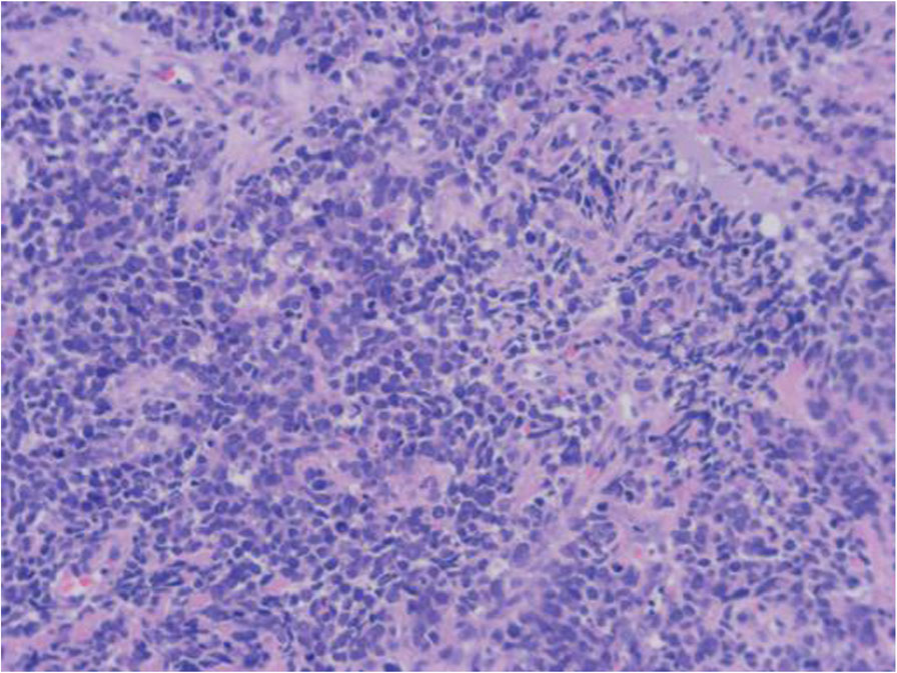
The tumor tissue is separated by fibrovascular vessels to form an epithelial patchy-like structure

**Fig. 2:**
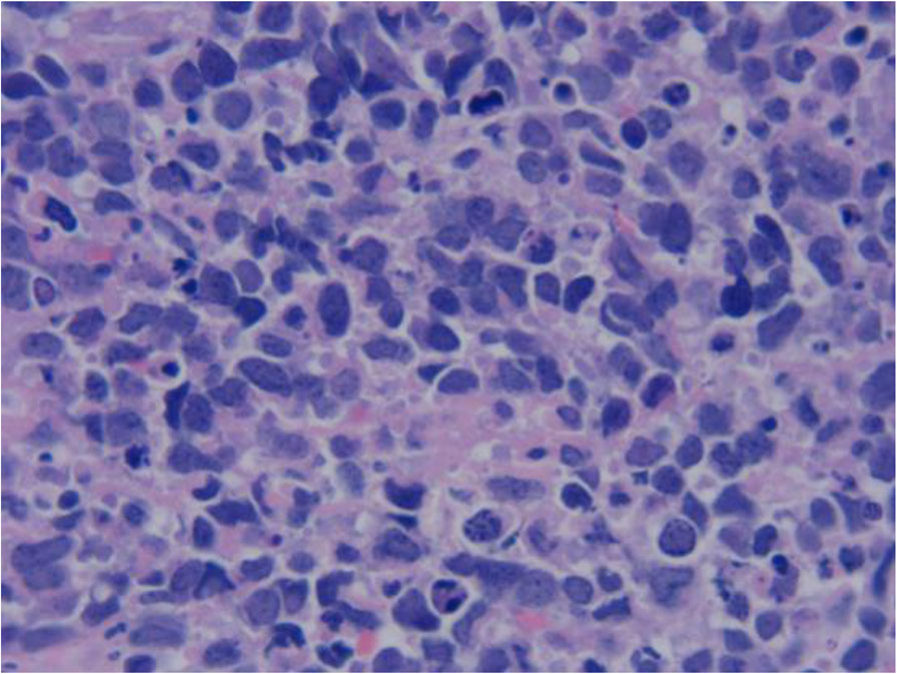
On cytological examination, the exhibits reduced cytoplasm; the cytoplasm contains the nucleus on one side with red-stained protrusions, at the defect site with invagination and red staining, or with red stains in the area surrounding the nucleus and clear boundaries

**Fig. 3:**
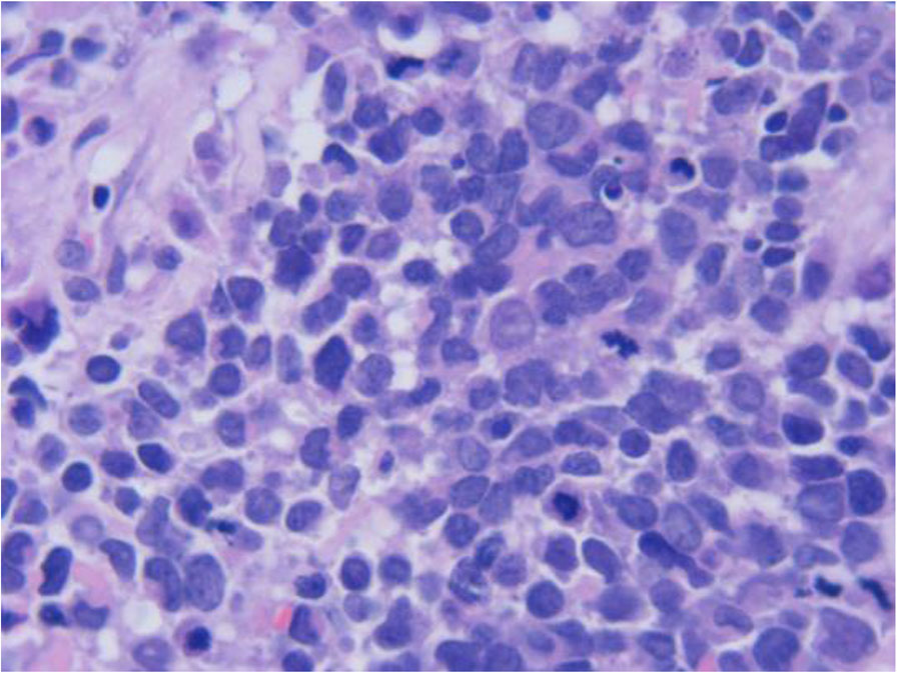
Fine nuclear chromatin can be observed with small nucleoli; moreover, the tumor cells are strongly eosinophilic with large nucleoli

**Fig. 4:**
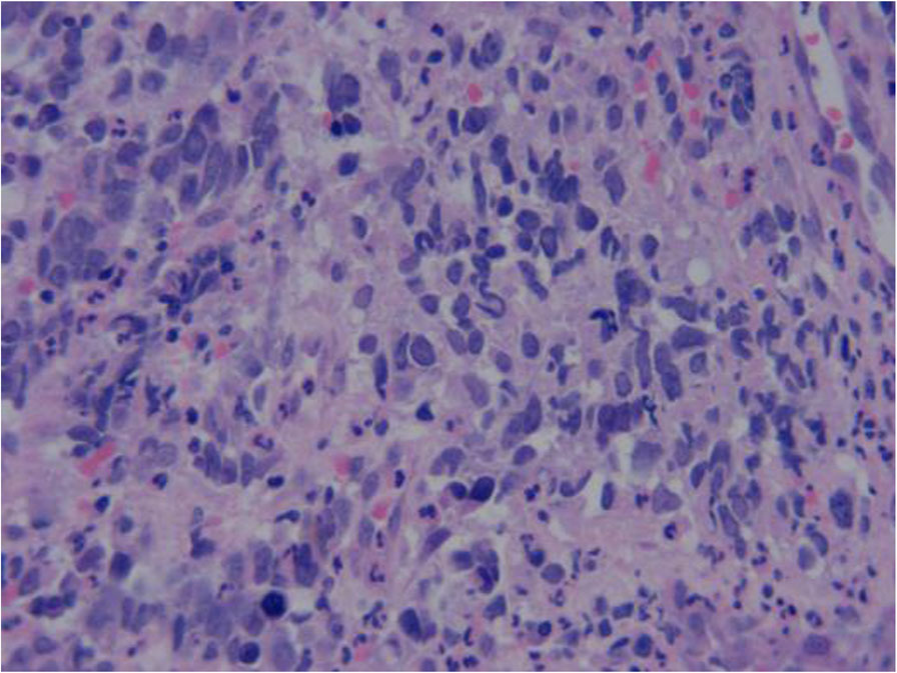
In some areas, as the cells differentiate, the acidophilic nature of the cytoplasm gradually increases. The cells appear fusiform, banded, or tadpole-like

**Fig. 5:**
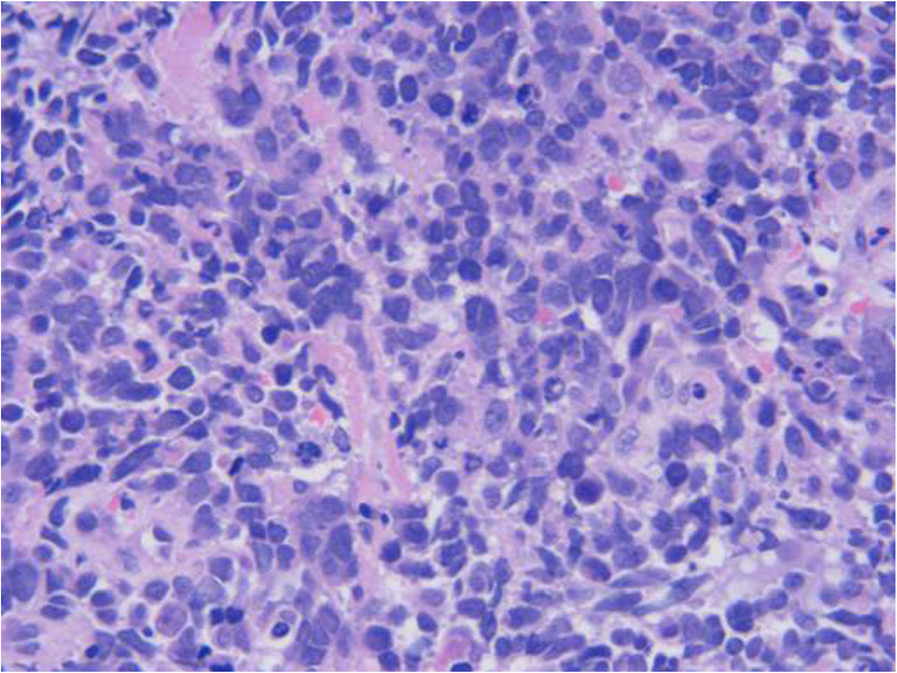
There is patchy necrosis. Characteristic, strip-like red-stained bands can be seen between the necrotic areas or between the tumor cells

A 64-year-old woman came to the Outpatient Department of Shenzhen Hospital of Southern Medical University with numbness and weakness in the limbs. Physical examination was negative except for slightly increased muscle tone in the right upper limb. Chest computed tomography (CT) revealed a thickening at the esophagogastric junction, extending over a length of 75 mm, with maximum thickness of ~ 16 mm. Abdominal CT showed enlarged lymph nodes at the bilateral posterior crura diaphragmatis, porta hepatis, and portal cavity interval, and also adjacent to the left gastric blood vessels, coeliac trunk, and abdominal aorta. Gastroscopy, revealed a friable mass bulging into the gastric cavity. The surface was markedly necrotic and bled easily on touch. The mucosa around the mass was congested and edematous, and the gastric angle was distorted. The gross appearance was suggestive of gastric cancer. Biopsy was obtained from three sites and sent for pathological examination. Microscopic examination showed necrosis and ulceration of the mucosa. The tumor was mainly composed of round cells of varying size, with the smaller ones resembling lymphocytes and the larger ones being 3–9 times the size of the smaller ones. Intervening fibrovascular septa created a patchy pattern (Fig. 1). Tumor cells had low cytoplasmic content and eccentric nuclei with fine chromatin and small nucleoli; there was red staining on one side of the nuclei (Fig. 2). About 10% of the tumor cells had strongly eosinophilic, large nucleoli, with clearly visible apoptotic bodies (Fig. 3). In some areas, cell morphology was markedly different: the tumor cells were fusiform, banded, tadpole-shaped, or giant sized, and the cytoplasm was more eosinophilic (Fig. 4). Furthermore, there was patchy necrosis, with strip-like red-stained bands in the necrotic area or between the tumor cells (Fig. 5). There were 5–9 mitotic figures per high-powered field (HPF).

**Fig. 6:**
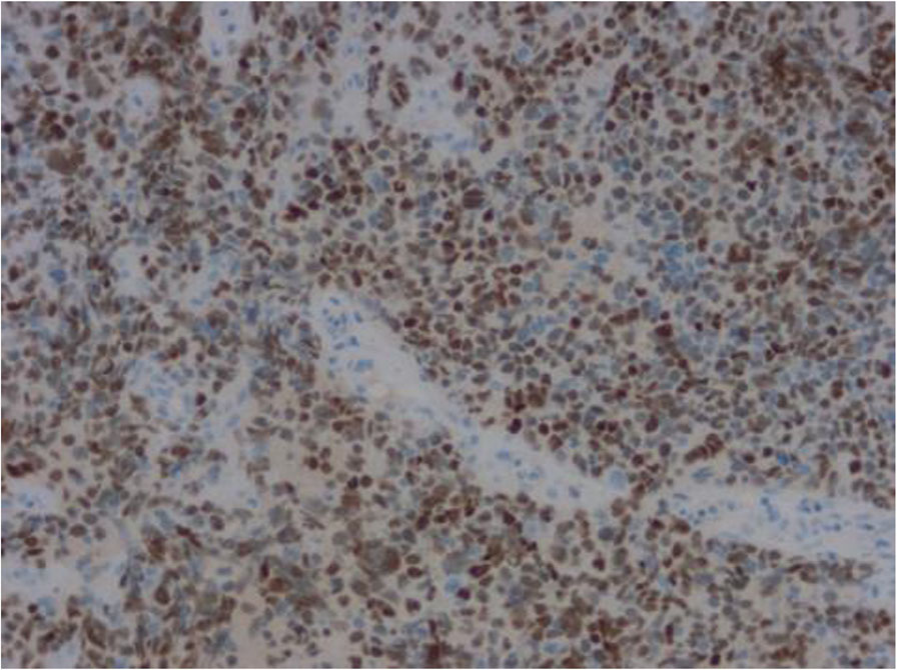
En Vision method shows strong positive expression of MyoD1

**Fig. 7:**
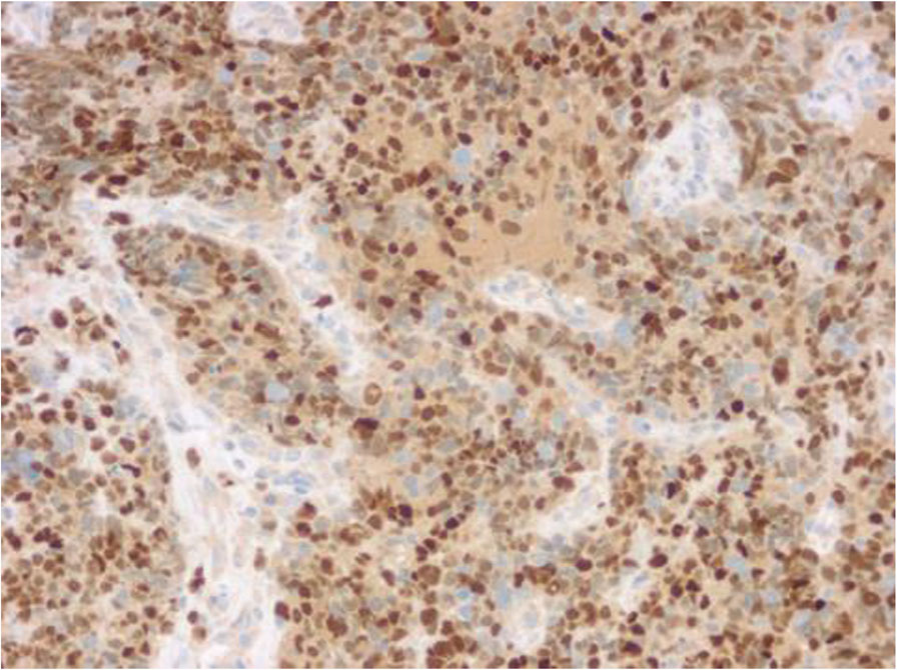
En Vision method shows strong positive expression of myogenin

**Fig. 8:**
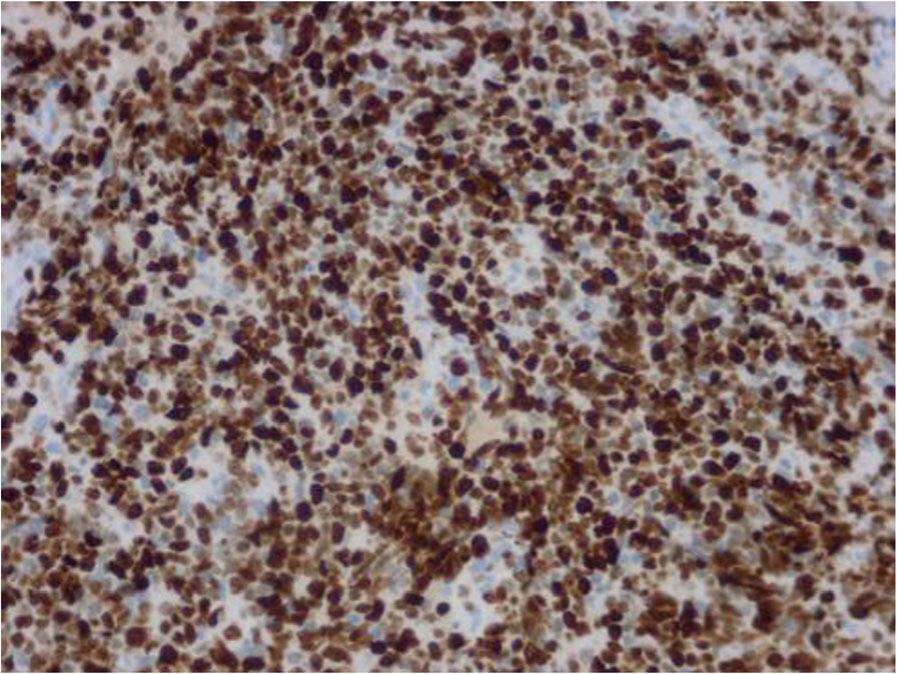
En Vision method shows ≥90% ki-67-positive cells

On immunohistochemical analysis the tumor cells were strongly positive for MyoD1 (Fig. 6), myogenin (Fig. 7), and the neuroendocrine cell marker PGP9.5, and weakly positive for desmin, actin, vimentin, CD56, and Syn. There was scattered positivity for epithelial cell markers CKpan and EMA. Staining was negative for CgA, S-100, HMB45, CD99, CD20, CD79a, CD30, ALK, CD117, DOG1, MUM1, FLI1, LCA, Bcl-2, TdT, and CD34. The ki-67 index was ≥90% (Fig. 8).

Based on these findings the patient was diagnosed with ERMS of the esophagogastric junction and was advised surgery. At surgery, patchy necrosis was seen in the overlying mucosa. The tumor was 2.6 mm in size and confined to the mucosa and lamina propria.

## Discussion and conclusion

Epithelioid rhabdomyosarcoma is a recently described variant and has not yet been included in the WHO classification, which currently recognizes four types: embryonal, alveolar, spindle cell, and pleomorphic. Embryonal rhabdomyosarcoma accounts for 60% of cases in children and occurs mostly in the genitourinary or head and neck regions. Histologic examination shows a mixed population of small round tumor cells with hyperchromatic nuclei and large polygonal-shaped tumor cells with abundant eosinophilic cytoplasm. The alveolar subtype accounts for 31% of all cases of rhabdomyosarcoma. It is most frequently seen in adolescents, occurring mainly in the extremities, trunk, or perianal regions. Histological examination shows a uniform population of intensely eosinophilic cells with a high nuclear–cytoplasmic ratio. The cells are sometimes loosely dispersed and mimic a pulmonary alveolar pattern. Fibrous tissue septa run through the tumor, creating “nests” of tumor cells. Spindle cell rhabdomyosarcoma is a subtype of embryonal rhabdomyosarcoma and accounts for 3% of all cases. It occurs mostly in the paratesticular region. These tumors show a spindled and leiomyomatous growth pattern. Marked rhabdomyoblastic differentiation and collagen deposition may be present. Pleomorphic rhabdomyosarcoma (or anaplastic rhabdomyosarcoma) is the least common of the four subtypes. It most often occurs in the 30–50-years age-group. Under the microscope, tumor cells typically show large, lobate hyperchromatic nuclei and multipolar mitotic figures.

The recently identified epithelioid rhabdomyosarcoma is characterized by an epithelioid morphology similar to that seen in poorly differentiated carcinoma or melanoma [4–6]. Only seven cases of ERMS have been reported in the literature to date. The patients included five males and two females, with a mean age of 56 years. The tumor arose in somatic soft tissue in four cases and in the organs in two cases [7]. Primary rhabdomyosarcoma of the stomach is extremely unusual. While there have been previous reports of gastric pleomorphic and embryonal rhabdomyosarcomas, we are the first to report a case of primary gastric ERMS [8, 9]. The histopathological features that we have described are consistent with those reported for ERMS at other sites. Cytokeratins are primarily makers for carcinomas; however, they are also frequently expressed in many sarcomas. Cell transformation during growth and differentiation of ERMS is accompanied by change in the immune phenotype. The aberrant cytokeratin positivity that develops is important for identification of ERMS.

Neuroendocrine carcinoma consists of relatively uniform small-to-medium-sized cells, with unclear cytoplasmic boundaries and round regular nuclei. Tumor cells may be arranged in beam-like, nested, or flaky patterns. Often multiple foci are present. Areas of necrosis and presence of > 2 mitotic figures/HPF are highly indicative of malignancy Tumor cells are positive for CgA, Syn, CD56, PGP9.5, and NSE, but negative for MyoD1, myogenin, myoglobin, and desmin [10].

Poorly differentiated adenocarcinoma of the stomach usually affects individuals in the age-group of 30–40 years. Clinical symptoms include weight loss and loss of appetite. Biopsy reveals small tumor cells with a loose glandular nest structure; occasionally there may be fusiform or irregular cancer cells. There may be 1–5 mitotic figures/HPF. The tumor is positive for CKpan, CK8/18, CK19, CK20, EMA, and CEA but negative for MyoD1, myogenin, myoglobin, desmin, and actin [11, 12].

Gastric epithelioid malignant melanoma is usually located in the lamina propria of the mucosa in the early stages. The tumor shows an adenoid, solid, nest-like structure, with little fibrous connective tissue. Tumor cells have richly basophilic cytoplasm with numerous melanin particles. A large, strongly eosinophilic nucleolus occupies ≥80% of the nucleus. These tumors are strongly positive for HMB45 and MART-1, but negative for MyoD1, myogenin, myoglobin, desmin, and actin [13–15].

Gastrointestinal stromal tumor (GIST) is the most common mesenchymal tumor of the gastrointestinal tract. They commonly develop in the elderly (age range, 60–65 years). Gastric GIST can develop at any site in the stomach. Histological appearance varies; the spindle cell type is the most common, followed by the epithelioid cell type. The pleomorphic cell type exhibits sarcomatoid characteristics, with a large number of atypical nuclei and mitotic figures. Most gastric GISTs are positive for CD117 and DOG1, and partially positive for CD34 and S1–00. They are negative for MyoD1, myogenin, myoglobin, desmin, and actin [16].

Primitive neuroectodermal tumor is composed of undifferentiated or poorly differentiated neuroepithelial cells. Microscopy shows small round tumor cells with little cytoplasm and deeply staining, chromatin-rich nuclei. Mitotic figures may be present. Single-cell necrosis is common. Tumor cells are positive for CD99, vimentin, GFAP, and NF, but negative for MyoD1, myogenin, myoglobin, and desmin [17].

Plasmablastic lymphoma is mostly composed of large cells resembling B immunoblasts, but the morphology may vary widely. The tumor cells form nested, adenoid structures in some areas. Mitotic figures are present, as also scattered multinucleated giant cells and macrophages that phagocytose dyeable small bodies. Large necrotic areas may be present. Tumor cells are positive for positive CD138, CD38, and IRP4/MUM1. About 50–80% of cases are positive for CD79a. There is no expression of MyoD1, myogenin, myoglobin, desmin, or actin [18].

Myeloid sarcoma in the stomach is a localized tumor formed by extramedullary proliferation and infiltration of myeloid primordial cells or immature myeloid cells. CT may reveal a mass in the gastric antrum. Patients often present with abdominal pain, melena, vomiting, and jaundice. MS is classified by the cell type involved. Granulocyte sarcoma, which is the most common, is composed of myeloblasts, neutrophils, and granulocyte precursor cells. These tumors are positive for MPO, lysozyme, CD68, and CD117, but negative for CD3, MyoD1, myogenin, and myoglobin [19].

To conclude, we report an extremely rare case of primary gastrointestinal ERMS. Better awareness of its features and the differential diagnoses will help in early diagnosis and treatment.

## Data Availability

The authors confirm that the data supporting the findings of this study are available within the article.

## Abbreviations

ALCL: Anaplastic large cell lymphoma
CT: Computed tomography
ERMS: Epithelioid rhabdomyosarcoma
GIST: Gastrointestinal stromal tumor
HPF: High-powered field
WHO: World Health Organization
